# The cost of systemic therapy for metastatic colorectal carcinoma in Slovenia: discrepancy analysis between cost and reimbursement

**DOI:** 10.2478/raon-2014-0046

**Published:** 2015-03-25

**Authors:** Tanja Mesti, Biljana Mileva Boshkoska, Mitja Kos, Metka Tekavčič, Janja Ocvirk

**Affiliations:** 1 Department of Medical Oncology, Institute of Oncology Ljubljana, Ljubljana, Slovenia; 2 Faculty of information studies, Novo mesto, Slovenia; 3 Faculty of Pharmacy, University of Ljubljana, Ljubljana, Slovenia; 4 Faculty of Economics, University of Ljubljana, Ljubljana, Slovenia

**Keywords:** cost of treatment, metastatic colorectal cancer, cost of targeted therapy, monitoring costs

## Abstract

**Background.:**

The aim of the study was to estimate the direct medical costs of metastatic colorectal cancer (mCRC) treated at the Institute of Oncology Ljubljana and to question the healthcare payment system in Slovenia.

**Methods.:**

Using an internal patient database, the costs of mCRC patients were estimated in 2009 by examining (1) mCRC direct medical related costs, and (2) the cost difference between payment received by Slovenian health insurance and actual mCRC costs. Costs were analysed in the treatment phase of the disease by assessing the direct medical costs of hospital treatment with systemic therapy together with hospital treatment of side effects, without assessing radiotherapy or surgical treatment. Follow-up costs, indirect medical costs, and nonmedical costs were not included.

**Results.:**

A total of 209 mCRC patients met all eligibility criteria. The direct medical costs of mCRC hospitalization with systemic therapy in Slovenia for 2009 were estimated as the cost of medications (cost of systemic therapy + cost of drugs for premedication) + labor cost (the cost of carrying out systemic treatment) + cost of lab tests + cost of imaging tests + KRAS testing cost + cost of hospital treatment due to side effects of mCRC treatment, and amounted to €3,914,697. The difference between the cost paid by health insurance and actual costs, estimated as direct medical costs of hospitalization of mCRC patients treated with systemic therapy at the Institute of Oncology Ljubljana in 2009, was €1,900,757.80.

**Conclusions.:**

The costs paid to the Institute of Oncology Ljubljana by health insurance for treating mCRC with systemic therapy do not match the actual cost of treatment. In fact, the difference between the payment and the actual cost estimated as direct medical costs of hospitalization of mCRC patients treated with systemic therapy at the Institute of Oncology Ljubljana in 2009 was €1,900,757.80. The model Australian Refined Diagnosis Related Groups (AR-DRG) for cost assessment in oncology being currently used is probably one of the reasons for the discrepancy between pay-outs and actual costs. We propose new method for more precise cost assessment in oncology.

## Introduction

Colorectal cancer is one of the most common cancers in the developed world. Morbidity and mortality caused by this form of cancer are increasing in Slovenia. In 2009 1,568 people were diagnosed with colorectal cancer.^1^,^2^ The increasing incidence also corresponds to increasing mortality because 50 to 60% of cases of the disease are discovered in an advanced stage, of which 20 to 30% have metastasized.

Patients in Slovenia with metastasized colorectal cancer (mCRC) that are physically or medically capable are mostly treated at the Institute of Oncology Ljubljana, where treatment takes place following guidelines adopted in line with globally recognized oncological guidelines, although the combinations of medications vary. 3,4

The costs of treating mCRC have risen quickly over the past decade, especially with the introduction of targeted medications for treating mCRC. With the introduction of the new targeted medications cetuximab and bevacizumab to mCRC treatment, the costs of standard care per person have increased from $500 to $250,000.^5^

There are no studies of defining mCRC treatment costs in Slovenia, and so we decided to take the first step and calculate what mCRC treatment costs amount to. For our analysis, we collected the costs of active patient mCRC treatment with systemic therapy at the Institute of Oncology Ljubljana in 2009 and compared them with payments by the Health Insurance Institute of Slovenia (ZZZS) with the goal to show if there is any discrepancy. The Institute of Oncology Ljubljana was the only healthcare facility in Slovenia where treatment of mCRC was carried out.

## Patients and methods

First we used retrospective analysis from an inventory of patient diseases and defined the database of treatment for patients treated at the Institute of Oncology Ljubljana in 2009 with systemic therapy for mCRC, and then we carried out a retrospective analysis of average *direct medical costs* of patient treatment for mCRC with systemic therapy from the *perspective of the hospital.* The study was approved by the institutional review board committee and was according the Declaration of Helsinki.

The study included costs of acute hospital procedures, whereas costs of non-acute hospital procedures were excluded.

For individual patients we took into account the information from the database shown in Table 1.

### Group characteristics

We analysed a homogenous group of 294 patients that underwent hospital treatment with systemic therapy. From the group of 409 mCRC patients that were treated at the Institute of Oncology Ljubljana, we excluded patients that took part in additional forms of treatment such as surgical procedures and radiotherapy (internal data from the gastrointestinal cancer team, Institute of Oncology Ljubljana) and 115 patients treated with systemic therapy only as outpatients, because evaluating the costs of outpatient therapy does not take place in the same way as evaluating the costs of hospital treatment. Evaluation of outpatient services takes place according to a point system (the Uniform List of Health Services, or green book), and hospital services are evaluated according to the diagnosis-related group (DRG) system.

The group included 123 men and 86 women. Approximately two-thirds (66%) were under 65-year old. Approximately 60% of patients (122) had primary localization of the tumour in the colon, and one-third in the rectum (69). The most frequent localization of metastasis was the liver (125 patients). One-fifth of patients had metastasis in the lungs or simultaneously in the lungs and liver, local recurrence of disease was present in nine patients, and the remaining one-third of patients had metastasis in the lymph nodes, bones, or pancreas and peritoneal carcinoma.

**FIGURE 1.**
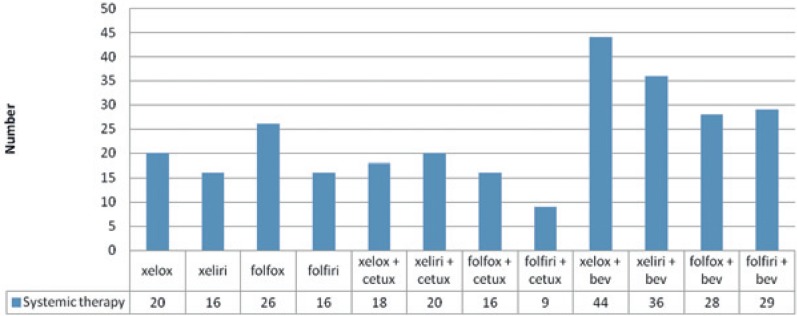
Distribution of systemic therapy.

The patients were treated with standard combinations of systemic therapy. Most patients received XELOX (capecitabine, oxaliplatin) + bevacizumab, which is understandable considering that capecitabine as a *per oral* form of fluoropyrimidine offers better quality of life for patients and visits to an oncologist are at three-week intervals, in contrast to 5-fluorouracil, which is an infusion form of fluoropyrimidine that is applied in 46 h infusions every two weeks. Paired chemotherapy with cetuximab was received by half as many patients as paired chemotherapy with bevacizumab, primarily because of the presence of KRAS mutation in the primary cancer tissue. The distribution of systemic therapy is presented in Figure 1.

### Definition of medications and procedures

Chemotherapeutics included fluoropyrimidine (5-fluororacil, capecitabine), irinotecan, and oxaliplatin, which we used in various regimens (fluorouracil, leucovorin, oxaliplatin, irinotecan [FOLFIRI], infusional fluorouracil, leucovorin, oxaliplatin [FOLFOX], capecitabine, irinotecan [XELIRI], capecitabine, oxaliplatin [XELOX]) and in combination with targeted medications: cetuximab, bevacizumab.^6^ The premedication included the following medications: dexamethasone 20 mg, granisetron 1 mg, and clemastine 2 mg. In the hydration we used 0.9% NaCl 2,000 ml or 5% glucose 2,000 ml (for only chemotherapy), or 0.9% NaCl 2,700 ml or 5% glucose 2,700 ml (for chemotherapy + targeted medication). Laboratory tests were divided into standard tests - a complete blood count (CBC), blood differential test, liver function tests, kidney function tests (nitrogen retention), electrolytes, C-reactive protein (CRP), and tumour markers (Carcinoembryonic antigen [CEA], carbohydrate antigen [CA 19–9]) and additional Laboratory tests that we carried out as needed: urinalysis, iron, ferritin, and transferrin. The imaging tests we included were: X-ray (lungs, abdomen, spine, pelvis), CT (thoracic cavity, abdomen), MRI (liver, lesser pelvis, head), bone scintigraphy, abdominal ultrasound, and PET-CT.

### Definition of costs

Because direct medical costs are fixed costs and variable costs that are directly connected to health condition or health treatment, in this analysis we defined direct medical costs as the sum of the following costs per patient per hospitalization (Table 2):^7^

- Cost of medications (cost of systemic therapy + cost of medications for premedication and hydration);
- Cost of labor to carry out systemic treatment (cost of labor per application + cost during time of hospitalization);
- Cost of lab tests carried out;
- Cost of imaging tests carried out;
- Cost of molecular test: defining KRAS mutation;
- Cost of hospital treatment due to side effects of mCRC treatment.

## Results

### Total direct medical costs of mCRC hospital treatment with systemic therapy in 2009

In 2009 the direct medical costs for mCRC hospital treatment with systemic therapy amounted to €3,914,697.00.

Direct medical costs for systemic therapy (chemotherapy + targeted medication) and medication for premedication and hydration amounted to €2,927,679.70.

Direct medical costs for laboratory tests amounted to €50,736.14, and direct medical costs for imaging tests €160,050.45.

Costs for testing for the presence of KRAS mutations amounted to €32,026.19.

Costs for the labor of applying medications amounted to €262,142.96, and costs for labor during the time of hospitalization were €733,110.64, which means that the cost of labor for carrying out systemic treatment amounted to €995,253.60.

Costs for hospital treatment due to side effects of systemic therapy amounted to €25,668.50. Only nine patients were treated, with an average length of hospital treatment of 12.9 days. The most common reason was diarrhoea (five patients), followed by sepsis without neutropenia (three patients). One patient had an allergic reaction to cetuximab. The reason there was such a low number of patients included in hospital care for side effects of systemic therapy is the good premedication and support therapy that the patients receive alongside systemic therapy, and especially hospital treatment of side effects of systemic therapy at specialized healthcare facilities.

**FIGURE 2.**
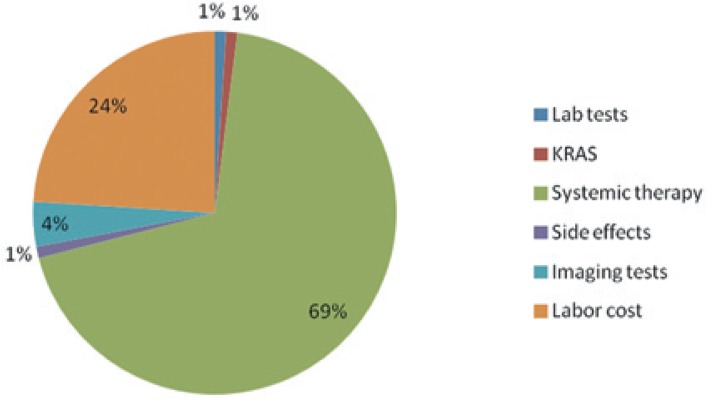
Distribution of direct medical costs (%).

The distribution of all direct medical costs is presented in Figure 2. Approximately seven-tenths (69%) of all direct medical costs were systemic therapy costs, which was also expected. One-fourth of the costs were the cost of carrying out systemic treatment. The greatest costs in the group of laboratory test costs were due to standard laboratory tests (84%). Thirteen percent of laboratory test costs were due to determining levels of iron, ferritin, and transferrin, and only 3% due to urinalysis.

**FIGURE 3.**
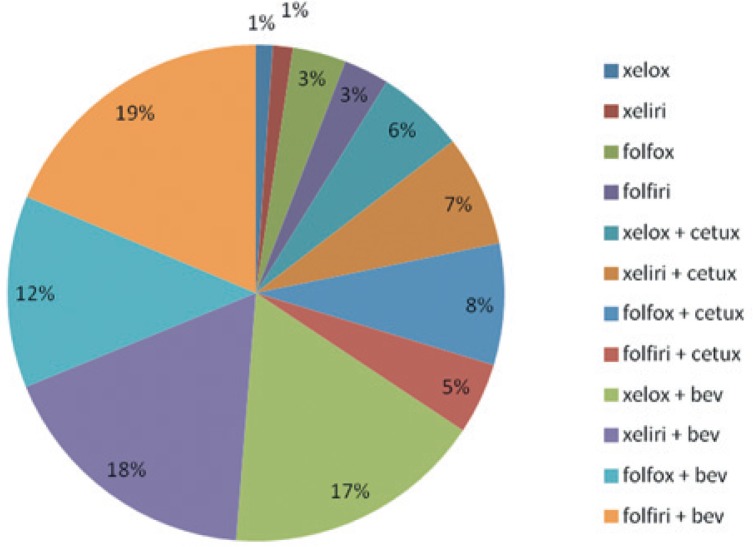
Distribution of systemic therapy costs by regimen (%).

With regard to percentages, the greatest costs were for combined systemic treatment; specifically, for chemotherapy in combination with bevacizumab, which was the most frequently used systemic mCRC treatment in 2009. Individually, out of all costs of medications for systemic treatment (chemotherapy and targeted medications), 19% of all costs were incurred for paired chemotherapy using FOLFIRI in combination with bevacizumab, 18% using XELIRI in combination with bevacizumab, and 17% using XELOX in combination with bevacizumab (Figure 3).

**FIGURE 4.**
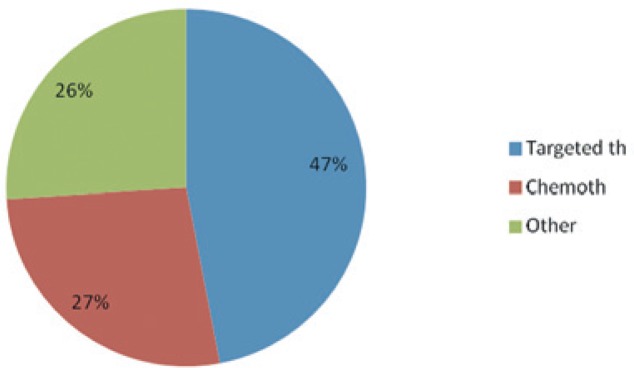
Proportion of targeted therapy costs to other costs

Targeted medications (cetuximab/bevacizumab) represent approximately 50% of the overall direct costs (€1,851,003.30), and the cost of chemotherapy is about 30% of all direct medical costs (€1,047,680.60). All other costs (laboratory tests, imaging tests, labor costs, costs of KRAS testing, and hospital treatment for side effects of systemic treatment) amount to 27% of direct medical costs, mostly due to the labor cost for carrying out systemic treatment (Figure 4).

### Average direct medical costs of hospital mCRC therapy with systemic therapy in 2009 per hospitalization

In the group of 209 patients treated with systemic therapy in 2009, altogether *1,605 hospital procedures* were carried out, and on average a patient was hospitalized 7.67 times.

Average direct medical costs of systemic treatment amounted to €2,439.10 per hospitalization.

The average costs of systemic therapy amounted to €1,806.00 per hospitalization

The average labor cost for carrying out systemic treatment amounted to €620.10 per hospitalisation.

Altogether, there were 1,629 standard laboratory tests and 1,547 additional lab tests (urine: 840, iron, ferritin, transferrin: 707) and 600 imaging tests (x-ray: 252, CT: 220, US: 59, MRI: 38, PET CT: 31, bone scintigraphy: 10).

The average cost for laboratory tests amounted to €15.97 per test, and for imaging tests €266.75 per test. On average, 1.98 lab tests and 0.37 imaging tests were conducted per hospitalization.

The average cost of systemic treatment amounted to €18,730.60 per patient.

The average cost of systemic treatment regardless of hospitalization but with regard to the number of rounds of systemic therapy received (two hospitalizations are necessary for one round with a combination of systemic therapy that includes 5-FU amounted to €3,323.17 per patient per round. Altogether, 1,178 rounds of systemic therapy were received.

### Comparison of hospital treatment costs recognized by the ZZZS with direct medical costs of hospital treatment of mCRC at the Institute of Oncology Ljubljana in 2009

The average value of DRG weights was 1.12 with a value of €2,739.63 for patients treated for mCRC with systemic therapy in 2009 at the Institute of Oncology Ljubljana (altogether there were 910 DRG cases). On average, each patient was hospitalized 4.35 times. Among the group of DRG cases that were present less than 10% in the calculation, there were 68, with a total weight number of 113.10 and an average value weight of 1.66.

The average value of one DRG weight for 2009 at the Institute of Oncology Ljubljana was €2,446.10.

The value of one DRG weight at the Institute of Oncology Ljubljana is higher in comparison with other providers of secondary activity because tertiary activity is also carried out.

The basic value of one DRG weight (for secondary treatment without added value for tertiary) amounted to €1,976.00 in 2009, which means that in 2009 the ZZZS paid the Institute of Oncology Ljubljana around €2,013,939.20 (if the average value of the DRG weight is 1.12) for patients that received hospital treatment for mCRC with systemic therapy.

*Direct medical costs* of hospital treatment for mCRC with systemic therapy at the Institute of Oncology Ljubljana in 2009, estimated as the cost of medications (cost of systemic therapy + cost of medications for premedication) + labor cost (cost of carrying out systemic treatment) + cost of lab tests performed + cost of imaging tests performed + cost of KRAS testing + cost of hospital treatment due to side effects of mCRC treatment, amounted to €3,914,697.

The difference between paid and actual costs, estimated as the direct medical costs of hospital treatment for mCRC with systemic therapy at the Institute of Oncology Ljubljana, was €1,900,757.80 in 2009.

The estimate and actual value of costs for hospital treatment of mCRC with systemic therapy at the Institute of Oncology Ljubljana in 2009 is presented in Table 3.

The average value of a DRG case amounted to around €2,213.12. Direct medical costs per hospitalization amounted to €2,439.10. Direct medical costs per patient amounted to €18,730.60.

## Discussion

### Costs of treating colorectal cancer

The most data on costs of treating metastatic cancers are provided by the United States, which have a profit-oriented healthcare system. This trend is also on the rise in Europe.

Analysis of the costs of mCRC, which are defined as the entire costs of the disease together with costs of treatment (costs during diagnosis, treatment, and follow-up) for a group of 6,746 mCRC patients treated between 2004 and 2009 showed that 52.2% of costs were incurred because of hospital treatment of patients, 22.2% of these because of surgical treatment and 47.7% because of out-patient treatment, 10.6% of costs connected with mCRC were incurred because of chemotherapy, and 11.1% were due to targeted medications.^8^

Costs connected with mCRC were defined as the percentage share of total costs and also contained the cost of chemotherapy and targeted medications (cetuximab, panitumumab, and bevacizumab). The cost of chemotherapy rose from 6.9% of total costs in 2004 to 8.1% in 2008. The cost of targeted medications rose from 4.8% in 2004 to 9.4% in 2008. Costs connected with mCRC were $9,978 per month. It is interesting that costs in the mCRC treatment phase were the lowest. The most costs were in the death phase ($26,649), followed by the diagnostic phase ($16,340). Ferro *et al*. determined that there was growth in the total costs of mCRC treatment from 1996 onwards, specifically due to increased choice among possible medications. Targeted medications have increased the cost of treating mCRC by a full 340-fold.^9^

The main cause of the overall costs of mCRC treatment are hospital treatment of patients ($37,369) and outpatient treatment ($34,582), which include chemotherapy. Monthly costs in the diagnostic phase ($12,205) were similar to in the death phase ($13,328), and costs in the treatment phase were considerably lower ($4,722).^10^

Information from England, where Bending determined the direct costs of treating bowel cancer to the National Health Service, where they used the clinical path to determine screening costs, diagnostic costs, treatment costs, and follow-up costs, indicate that the entire annual costs of treating bowel cancer were approximately £1.1 billion. The greatest share of the costs were in the diagnostic phase (£291 million). Treatment costs defined as primary treatment costs (surgery and pharmacotherapy) were approximately £201 million, £129 million for primary treatment of colon cancer, and £72 million for treatment of colon cancer.^11^

In Slovenia, some oncology studies have been carried out that have determined cost effectiveness. Piškur *et al.* analysed the cost effectiveness of the hormone medications anastrozole and tamoxifen for breast cancer.^12^ Obradović *et al.* analysed the cost effectiveness of determining the UGT1A1 genotype for irinotecan in monotherapy for colorectal cancer.^13^

In Slovenia to date there has been no analysis that defines mCRC treatment costs, and therefore there are no data from comparable studies in Slovenia.

### Evaluating the costs of hospital treatment

In Slovenia the main share of healthcare expenditures are provided from public funds. In 2008 these expenditures comprised of public sources amounted to 72.3% of all funds.^14^

In 2004 Slovenia moved from financing hospitals on the basis of services (the criterion was the green book), to length of stay-based financing on the model of paying DRG to those providing specialized hospital activities. Acute hospital treatments in the DRG model are categorized by diagnoses and procedures performed according to the Australian modification of the tenth revision of the International Statistical Classification of Diseases and Related Health Problems (ICD-10-AM), adapted to Slovenian circumstances, and confirmed by the Health Council.

The DRG system is the most common payment method internationally. This is a payment system that was developed by a group of hospital administration experts at Yale University in the United States by setting up twenty-three main diagnostic groups containing 467 diagnoses. Since 1982, the DRG system has been used as a system for financing health services in the American Medicare program.^15^ Various countries have adopted the DRG system with adaptations to their national environments (Canada, Australia, the Scandinavian countries, France, Italy, Austria, and Germany). In Slovenia the DRG system was introduced in 2003 based on the model used in Australia (Australian Refined Diagnosis Related Groups, AR-DRG). The model is based on the principle of “the money follows the patient.” At the Institute of Oncology Ljubljana, as at all other hospitals carrying out acute hospital care, since 2004 assessment of health treatment has taken place following the DRG system. DRG are defined through diagnoses and procedures carried out following ICD-10-AM and other information such as length of treatment, age, sex, weight upon admission, and hours of mechanical ventilation.

The DRG system is essentially a method of classifying patients into groups based on different levels of demandingness for whom approximately the same funds are used. An individual acute hospital treatment for a patient is categorized into one DRG case. The price of an individual group of similar cases is expressed in relative terms, and is weighted with regard to price, or the price of an average case. The weight is expressed based on a coefficient of 1. More demanding or expensive DRGs have a weight greater than 1, and less demanding or cheaper ones have a weight less than 1. According to the current method, the ZZZS sends the price of one weight to all hospitals in Slovenia, on the basis of which the hospitals then calculate the services they carry out. Together with the weight of this DRG case and based on the price of one weight, they can calculate the price of a particular acute hospital treatment and, based on the weight of an individual case and the value of the weight, they can define the price of a case.

This method of evaluating hospital treatments— the DRG system or groups of comparable cases— obviously has its disadvantages. Disadvantages of the DRG model may include excessive reduction of costs by limiting necessary tests and choosing less appropriate medications, admitting patients that do not need hospitalization, and false representation of diagnoses and treatments with higher prices, or the provider adapting the data for greater profit with worse medical treatment.^16^ Obviously this is not the case for non-profit providers such as the Institute of Oncology Ljubljana, where direct medical costs are higher than those actually paid by the ZZZS.

In his master’s thesis, Jurij Stariha draws attention to the lack of uniformity in DRG weight for comparable groups of cases in various hospitals in Slovenia.^17^ In principle, for equivalent cases (i.e., cases in which the same amount of resources is used) hospitals should receive the same payment defined by the price of handling an individual case or the weight of the DRG case and the price of one weight. Stariha showed that in 2007 there was not a uniform weight for all providers, which means that providers received different payment for handling equivalent DRG cases. The confirmation that there is a lack of connection of price weight with indicators of operation shows that the current system of financing is not optimal, and so providers or hospitals adapt the value of the weight that they receive.

From the analysis of the DRG system by the Public Health Institute (2003–2008) it is also clear that the current system of financing is not the best and that the level of weight is in great need of adjustment.^18^

According to Marušič *et al*.^15^, in order to define standard costs of cases the improvement process ought to take into account clinical paths that precisely describe the handling of a particular case following evidence-based principles of modern medicine. For less frequent cases, actual average costs can be used. It is important to adapt statistical data by taking into account the relationship between the costs of work performed and the differences in length of hospital stay. The DRG model cannot represent all of the complexities of hospital organization. Likewise, a very complicated individual case cannot be classified such that the costs are rejected. In defining weighting, it is not possible to take into account the likelihood of very rare complications, which result in very high additional costs.

### Monitoring costs by business process activities as better model for cost assessment in oncology

Currently in Slovenia the AR-DRG 4.2 version of the classification system from 2000 is still being used, even though the updated AR-DRG 6.0 from 2008 (Australian Government, Department of Health and Ageing) is available.

The old version of AR-DRG being used in Slovenia is one of the reasons for the discrepancy between pay-outs and actual costs. Of course, the comparison is not optimal because this study did not include complete costs, but it can be concluded that the discrepancy would be even greater if it took into account the entire cost. Regardless of the version of the evaluation system, it is necessary to adapt the weighting to the actual costs. Oncology is an area of medicine that is developing and changing extremely quickly, which is shown in everyday news on treating cancer. One of the most dynamic areas in oncology in the last decade has been treating mCRC. Classifications and cost evaluation systems for treatments are quite rigid and changes in these areas take place slowly, which is reflected in non-objective evaluation of the costs of mCRC treatment.

Classifying and financing procedures following the DRG model should orient providers towards cost-effective treatment, but not also toward quality. Marušič, Ceglar, and Prevolnik-Rupel suggest including financing criteria such as user satisfaction and treatment outcome for the most common procedures. In this manner, the dimension of quality will be built into payment.^15^

The solution to the problem could be that which was proposed by the Public Health Institute in its DRG analysis (2003–2008): it is necessary to carry out a cost analysis among providers and to define the nationally acceptable average for cancer treatment. However, it is especially emphasized that that it is necessary to define oncology treatment at the Institute of Oncology Ljubljana as the oncology center in Slovenia where most patients with mCRC are treated as a separate entity. Specifically, the price of DRG in Slovenia is based on evaluating the cases of three pilot hospitals, which was carried out based on data on hospital costs in 2001: the Ljubljana Medical Center, Maribor General Hospital, and Jesenice General Hospital. In 2003, based on cost analysis data from the study of pilot hospitals and the Australian weights in the National Hospital Cost Data Collection Round 6 (2001–2002), weights were calculated and then the demandingness of individual cases was determined in Slovenia.^15^

Considering that in the costs we include every monetized use of business elements in achieving the effects of a business process, which in addition to costs in the narrowest sense also includes all elements that are deducted from the profits for a given period, the ideal cost system should ensure completely precise data on all costs with regard to all business effects that a company achieves.^19^ The costs, which should ensure one hundred percent reliable information, would far exceed the benefits because they would be too high. Because of this, a company must seek the optimal level of information precision, which can be achieved by monitoring costs by business process activities.

Monitoring costs by business process activities makes it possible to classify general costs and is based on constant improvement of individual parts of the business process; this therefore handles costs at the level of an individual activity, because the activity is the thing that creates the cost. The process of determining and monitoring costs by business process activities takes place such that it is necessary to first define the areas that will be included in defining the costs; for example, the costs of the overall oncological process (pre-hospital testing, pre-hospital care or screening and limiting tests, operation costs, anaesthesia, postoperative costs, material used for this, radiotherapy costs, systemic therapy costs) or for only one area of care. This is followed by defining sources included in analysing the costs that were defined in the previous stage. The third phase defines the specific activities carried out by each of the sources; for example, precisely defining the services that the internal oncology department carries out, a description of the services by staff in an individual department, and the time needed for a particular health service. In the final phase it is necessary to assign a cost to each part. For this we use direct costs, for example, for medicines, services such as lab tests, imaging tests, the material use costs using material, including consumables and technology used, staff, and total direct and indirect labor costs, including employee benefits.^20^

In this manner we can discover and eliminate all unnecessary activities in a healthcare organization so that those that are essential from the viewpoint of treatment and operations will be carried out with maximum efficiency. This will make it possible to achieve lower overall healthcare costs.

This analysis is based on a treatment reality of mCRC that has since remained virtually unchanged. Hence, it can be assumed that the results are still valid today.

## Conclussions

The costs paid to the Institute of Oncology Ljubljana by health insurance for treating mCRC with systemic therapy do not match the actual cost of treatment. In fact, the difference between the payment and the actual cost estimated as direct medical costs of hospitalization of mCRC patients treated with systemic therapy at the Institute of Oncology Ljubljana in 2009 was €1,900,757.80.

Classifications and cost evaluation systems for treatments are quite rigid and changes in these areas take place slowly, which is reflected in non-objective evaluation of the costs of mCRC treatment.

The model AR-DRG for cost assessment in oncology being currently used is probably one of the reasons for the discrepancy between pay-outs and actual costs. We propose new method for more precise cost assessment in oncology. Monitoring costs by business process activities makes it possible to classify general costs and is based on constant improvement of individual parts of the business process; this therefore handles costs at the level of an individual activity, because the activity is the thing that creates the cost.

We conclude that the only solution is establishing better communication between oncology, economics, and pharmacoeconomics, which also demands considerably greater transparency of data and accessibility, and only after this it is possible to develop a suitable model for defining costs in oncology.

## Figures and Tables

**TABLE 1.** Data in the database

**No.**	**Data**
1	Age
2	Sex
3	Number of hospital procedures for 2009
4	Localization of primary cancer: colon, rectum, colon-rectum transition
5	Localization of mCRC metastasis: liver, lungs, liver and lungs, local recurrence of disease, other
6	Line of treatment with systemic therapy in 2009: first, second, third, fourth, and number of lines of systemic therapy received by an individual patient in 2009 (one course, two or more lines)
7	Systemic therapy regimen: number of hospital applications and dose of individual medications in regimen and price of medicine
8	Number, dose, and price of medication for premedication per individual application
9	Number, dose, and price of medication for hydration per individual application
10	Laboratory tests carried out for an individual patient during hospital treatment due to systemic therapy, type, number, and price of lab tests
11	Imaging tests carried out for an individual patient during hospital treatment due to systemic therapy, type, number, and price of imaging tests
12	Hospital services per patient due to side effects of systemic therapy
13	KRAS testing before the start of treatment with systemic therapy for mCRC patients before the first line of therapy (yes/no)
14	*Labor* costs for carrying out systemic therapy per hospitalization

mCRC = metastatic colorectal cancer

**TABLE 2.** Definition of costs

**Costs**	**Definition**
**Medication^a^**	Medication cost = dose and number of applications of medication calculated according to medication supply price^b^
**Lab tests**	Test costs = (number of points^c^ of test + number of points scored (venous blood draw)) × cost price of a point^d^
**Imaging tests**	Test costs = number of points^c^ × cost price of a point^d^
**Applying medication**	Sum of the labor cost of nurses, physician, pharmacist, pharmaceutical technician, and administrative and technical staff with regard to the average time used for application, and average value of an hour of labor for an individual involved in applying medication.
**Work during hospitalization**	Sum of the labor cost of nurses, physician, average time used per patient, and average value of an hour of labor for an individual during hospitalization^e^
**Testing primary cancer or metastasis for KRAS mutation**	Cost of molecular analysis + labor cost
**Hospital treatment for side effects of systemic therapy**	Sum of the cost of medications used (parenteral antibiotics, peroral antibiotics, parenteral feeding, hydration, other medication), tests (lab and imaging), and labor during hospitalization.

a Excludes cost of Xeloda (capecitabine) because patients receive it with a prescription at an external pharmacy and then continue their therapy at home.

b Supply of medications at the Institute of Oncology Ljubljana takes place through a public procurement process as defined by law (Public procurement Act, *Official Gazette of the Republic of Slovenia*, no. 16/08).

c The green book or Uniform List of Health Services contains a point value for health services based on the need for staff and time used expressed in minutes for carrying out these services. It is a very old and outdated document that contains a description of all health exams, care, and tests with precise codes, description of health services and of staffing and time standards, whereby all services are evaluated with points. This document is still used in calculating health services performed and in checking billing accuracy even though many modern services are not included in it.

d The cost price of a point is defined retroactively for 2009 by individual diagnostic unit (Analysis of costs and physical indicators for 2009, Institute of Oncology Ljubljana) based on values from the green book. The cost price of a point per individual diagnostic unit represents the quotient between the total costs of an individual diagnostic unit for an individual year and the number of points realized.

e Systemic treatment that includes capecitabine (capecitabine, oxaliplatin [XELOX], capecitabine, irinotecan [XELIRI], XELOX/bevacizumab, XELIRI/bevacizumab, XELOX/cetuximab, XELIRI/ cetuximab) involves one-day hospitalization, and systemic treatment that includes 5-FU (infusional fluorouracil, leucovorin, oxaliplatin [FOLFOX], fluorouracil, leucovorin, oxaliplatin, irinotecan [FOLFIRI], FOLFOX/bevacizumab, FOLFIRI/bevacizumab, FOLFOX/cetuximab, FOLFIRI/cetuximab) involves three-day hospital treatment.

**TABLE 3.** Estimate and actual value of costs for 2009

**Cost estimate (2009)**	**Total cost (€)**	**DRG case / hospitalization (€)**	**DRG case / round of systematic treatment (€)**
Costs estimated by ZZZS	2,013,939.20	2,213.12	2,213.12
Direct medical costs	3,914,697.00	2,439.10	3,323.17
**Difference**	**1,900,757.80**	**225.98**	**1,110.1**

DRG = diagnosis-related group
